# A sharp plant stem causing terminal ileal perforation with clinical presentation resembling acute appendicitis: a case report

**DOI:** 10.3389/fmed.2025.1713022

**Published:** 2025-11-28

**Authors:** Pirada Yincharoen, Weeratian Tawanwongsri

**Affiliations:** 1 Department of Surgery, School of Medicine, Walailak University, Nakhon Si Thammarat, Thailand; 2 Department of Internal Medicine, School of Medicine, Walailak University, Nakhon Si Thammarat, Thailand

**Keywords:** foreign bodies, intestinal perforation, appendicitis, plants, abdominal pain, case report

## Abstract

**Introduction:**

Ingestion of plant material is an unusual cause of small-bowel perforation and can have a clinical appearance similar to acute appendicitis.

**Case description:**

A 40-year-old Thai man presented with a 1-day history of right lower quadrant pain and low-grade fever. Laboratory testing showed leukocytosis. Contrast-enhanced computed tomography (CT) demonstrated a mildly dilated appendix, 7 mm, without periappendiceal fat stranding, free air, abscess, or visible foreign body. Because localized peritonism persisted despite equivocal imaging, exploratory laparotomy via a Lanz incision was undertaken. A 2.5-cm, needle-like wooden fragment was palpated within the terminal ileum, causing a localized perforation; it was subsequently identified as the lateral stem of *Gnetum gnemon* var. *tenerum*, an edible plant. The fragment was removed, the ileal defect was closed primarily, and appendectomy was performed. The postoperative course was uncomplicated.

**Conclusion:**

This case illustrates the limitations of imaging in the case of small-bowel perforation, and contributes to the literature on plant material ingestion as a cause of acute abdomen in the context of regions where such foods are consumed.

## 1 Introduction

Small bowel perforation is an uncommon cause of acute abdomen, reported at roughly 0.4% of cases in one series and with an estimated incidence of 1 per 300,000–350,000 individuals (1). The condition carries substantial risk: in an Asian surgical cohort, the mortality was 19.1% and perioperative morbidity 57.4% (2). Outcomes are influenced by patient factors (advanced age, physiologic derangement such as hypotension, elevated C-reactive protein, or organ failure), etiology (e.g., malignancy, ischemia, or typhoid fever), nutritional status (hypoalbuminemia), and comorbidities, including chronic steroid use, dependence in activities of daily living (ADLs), and preoperative septic shock (3–5).

Clinical manifestations are often nonspecific—typically abrupt, persistent abdominal pain with peritoneal signs—and can rapidly progress to peritonitis and sepsis if not promptly managed (1). Because symptom patterns overlap with more prevalent conditions, diagnosis relies on integrating history, examination, and imaging, with appreciation that imaging may be nondiagnostic early in the course.

Causes span mechanical, inflammatory, infectious, neoplastic, and traumatic processes. In one institutional series, adhesions (20.3%), tumor implantation (16.9%), herniation (15.3%), and inflammatory bowel disease (10.2%) predominated, while foreign body impaction accounted for 6.9% of cases (6). Plant-derived foreign bodies represent a particularly rare subset and may be underrecognized in regions where fibrous edible plants are commonly consumed. We report a case of terminal ileal perforation caused by the lateral stem of *Gnetum gnemon* var. *tenerum* (“liang”), presenting with features mimicking acute appendicitis. This case highlights a dietary risk specific to local culinary practices and underscores diagnostic challenges when imaging is nondiagnostic.

## 2 Case description

A 40-year-old Thai male presented to the emergency department with a one-day history of dull, steady, right lower quadrant (RLQ) abdominal pain. He had an unremarkable medical and family history with no gastrointestinal symptoms prior to this episode. He described the pain as localized, non-radiating, and progressively worsening. A review of systems was positive for fever, but he denied nausea, vomiting, anorexia, diarrhea, constipation, or any urinary symptoms. The patient had no history of abdominal surgery or trauma. His last meal prior to symptoms was approximately 7 h ago. On admission, the patient was alert and hemodynamically stable. Vital signs were temperature 37.0 °C, blood pressure, 146/92 mmHg; pulse, 88 beats/min; respiratory rate, 20 breaths/min; and oxygen saturation, 100% on room air. Physical examination revealed a soft abdomen without distension, but there was marked localized tenderness in the RLQ, which was associated with rebound tenderness without guarding. The remaining examinations were unremarkable. Laboratory investigations showed a hemoglobin 13.3 g/dL, white blood cell count of 14,170/μL (71% neutrophils), and platelet count of 355,000/μL. Serum electrolyte levels, renal function, and coagulation profiles were within the normal limits. An acute abdominal series radiograph demonstrated no evidence of pneumoperitoneum, bowel obstruction, or other acute intra-abdominal pathologies. A contrast-enhanced CT scan of the abdomen revealed a mildly dilated appendix (7 mm in diameter) with no focal mass or periappendiceal fat stranding suggestive of early-stage appendicitis. No evidence of perforation, abscess, or foreign bodies was found. A detailed chronological timeline of the patient’s clinical course, investigations, treatment, and outcomes is provided in Supplementary File S1. Given localized peritonism despite equivocal imaging, operative exploration was undertaken.

**Figure 1:**
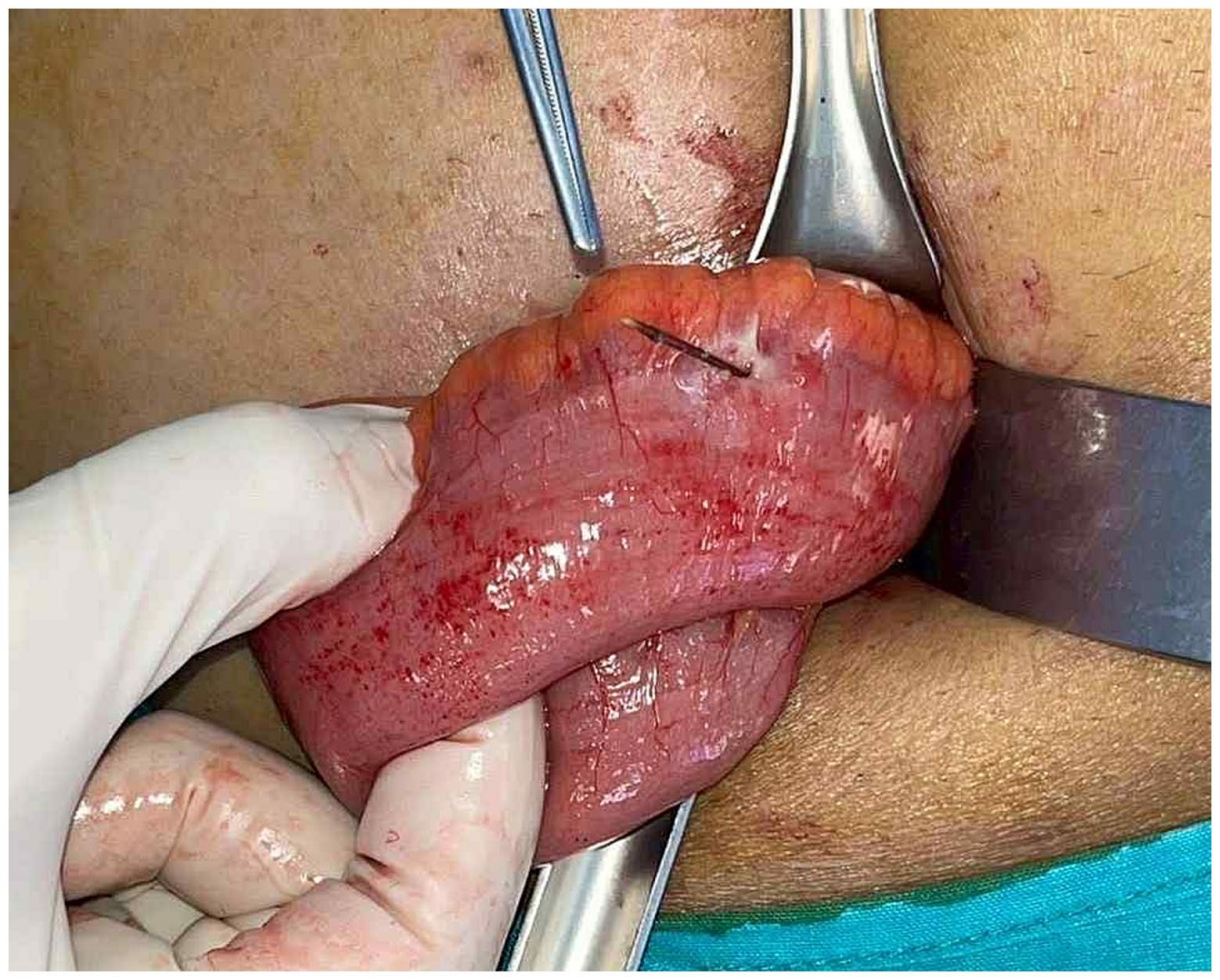
Intraoperative view showing a sharp wooden foreign body perforating the terminal ileum. The image was taken before completion of irrigation and contamination-control steps, including isolation of the affected segment with laparotomy pads.

On the day of admission, he underwent exploratory laparotomy through a Lanz incision. Digital exploration was initially performed to localize the appendix. However, during palpation, a firm wooden foreign body was unexpectedly identified within the terminal ileum. This, approximately 2.5 cm in length, caused a localized perforation (Figure 1). It was later identified as the lateral stem of *Gnetum gnemon* var. *tenerum*, a plant consumed locally as a vegetable. Intraoperatively, minimal reactive peritoneal fluid was observed around the perforation. The affected ileal segment was isolated with laparotomy pads, the fluid was suctioned, and the peritoneal cavity was irrigated with warm normal saline. Specimen handling was performed away from the incision. Perioperative antibiotics were administered according to protocol, and instrument and glove changes were performed prior to closure. The foreign body was removed and the perforated ileum was repaired with interrupted sutures. An appendectomy was performed and the specimen was sent for histopathology. No cecal mass was identified. Estimated blood loss was 10 mL. The patient tolerated the procedure and anesthesia well, resumed oral intake on postoperative day 2, and had an uncomplicated recovery without surgical-site infection. Histopathology of the appendix showed unremarkable mucosa with a fecalith in the lumen, slightly congested serosa, and submucosal lymphoid follicles with prominent germinal centers; there was no mucosal or muscular inflammation, and the serosa exhibited edema with mild neutrophilic infiltrates. He was discharged home and remained asymptomatic at the two-week follow-up.

## 3 Discussion

**Figure 2:**
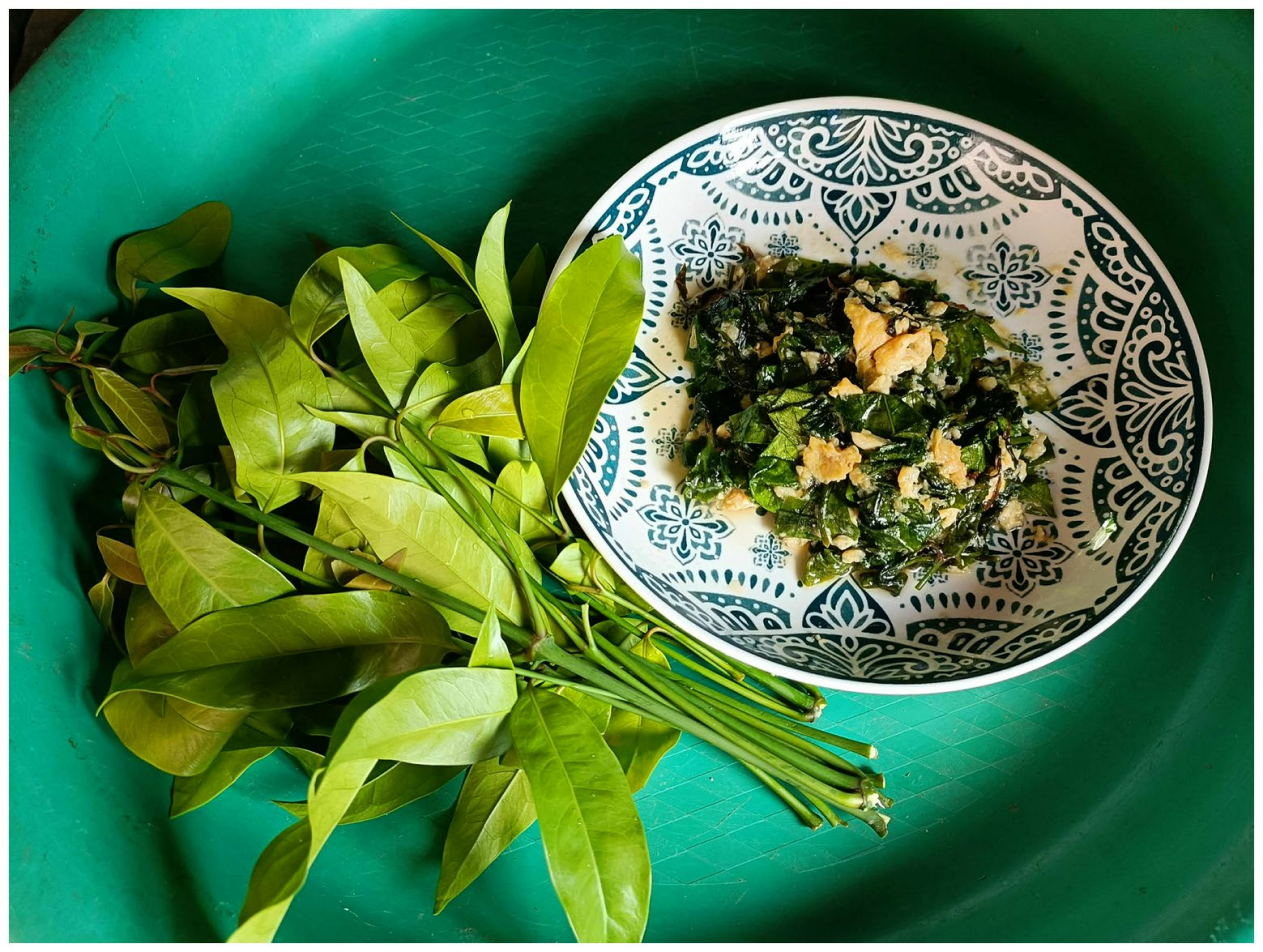
Fresh leaves of *Gnetum gnemon* var. *tenerum* (“Liang” in Thai) and a common local recipe of stir-fried Liang leaves and eggs. In local culinary practice, fresh leaves are used and preferred; the lateral stems are often discarded.

This case demonstrates an unusual etiology of acute abdominal pain, in which a fragment of a plant stem produced terminal ileal perforation that clinically mimicked acute appendicitis. There are, to our knowledge, no previous reports of small-bowel perforation due to *Gnetum gnemon* var. *tenerum* (Figure 2), suggesting that the species is a potentially confounding and unique dietary risk factor in endemic culinary contexts. The imaging demonstrated suggestive findings, albeit nonspecific; it was only intraoperatively when we identified the foreign body at the perforation site that we made the definitive diagnosis. Despite the suspicion of early-stage appendicitis, there was diagnostic uncertainty. In such presentations, many centers may choose diagnostic laparoscopy because it has demonstrated high diagnostic performance for appendicitis, with a sensitivity of 92% and an overall accuracy between 95 and 99%, and can reduce unnecessary appendectomies where the appendix is found to be histologically normal at surgery (7). Laparoscopy can help identify alternative intra-abdominal pathology in equivocal RLQ pain. Laparoscopy allows for immediate therapeutic intervention if appendicitis is confirmed (7, 8). Moreover, it is associated with smaller incisions, less postoperative pain, shorter length of stay, and fewer wound complications and adhesions compared to open methods (9). In this case, given localized peritonism and incongruent imaging, along with a need to evaluate both the appendix and terminal ileum, the team decided to proceed with immediate exploration via a Lanz incision, which allowed for organized evaluation, identification of the ileal perforation, definitive primary repair, and appendectomy for the fecalith. Our case highlights the clinician’s challenges of diagnosis and the importance of keeping a wide, anatomy-based differential diagnosis for patients with RLQ pain, especially when clinical and radiologic findings diverge.

We also reviewed the literature for plant-related foreign bodies with bowel perforation and summarized it in Table 1 to provide context for our observation. Two previously published cases of small-bowel perforation due to leaf and thread from plant-origin-derived material have a similar anatomic location and demonstrate a potential mechanism of injury. Mohanty et al. (10) presented a case of a 45-year-old man with 5 days of abdominal pain and decreased appetite, as well as a low-grade fever; imaging revealed free intraperitoneal air, and it was ultimately determined intraoperatively to be a 5-cm right-angle thorn from *Vachellia nilotica* perforating the terminal ileum and was corrected with primary repair with uneventful postoperative follow-up over 1 year, and Shafique et al. (11) described a 64-year-old woman with multiple comorbidities who presented with peritonitis from a seed bezoar. During laparotomy, a 2 cm perforation was found 8.5 feet distal to the duodenojejunal flexure with extensive gangrenous small bowel caused by an apricot, peach, mango pickle, and java plum seeds. Resection with loop ileostomy was performed; however, the patient died postoperatively due to cardiac arrhythmias. These cases, in addition to our case, demonstrate that plant materials may be a rare yet clinically significant cause of perforation of the small bowel.

**Table 1:** Reported cases and series of plant-origin foreign bodies associated with bowel perforation.

Authors (year)	Country	Study design	Patient characteristics	Causes	Clinical presentation	Bowel perforation site	Management and outcomes
Shafique et al. (2024) (11)	Pakistan	Case report	A 64-year-old female	Seed bezoar containing 1 apricot seed, 1 peach seed, 1 mango pickle seed, and 4 java plum seeds	Generalized abdominal pain and vomiting for 3 days; tachycardia, tachypnea, hypotension; tense, rigid abdomen with absent bowel sounds	Small bowel, approximately 8.5 feet distal to the duodenojejunal flexure	Emergency laparotomy after resuscitation; resection of gangrenous small bowel; loop ileostomy formation; intraoperative cardioversion for ventricular tachyarrhythmias; postoperative ICU care; patient died due to cardiac arrhythmias
Suárez-Gómez et al. (2024) (28)	Colombia	Case series	A 28-year-old male (the only vegetable foreign body case among five patients)	Vegetable foreign body	48-h history; peritoneal irritation; no fever; tachycardia (HR 108 bpm) with normal BP (110/70)	Meckel’s diverticulum	Resection and anastomosis with omental patch, incidental appendectomy; survived; favorable follow-up
Mohanty et al. (2023) (10)	India	Case report	A 45-year-old Caucasian male	Gum Arabic plant (*Vachellia nilotica*)	Severe generalized abdominal pain, low-grade fever, and reduced appetite for 5 days; abdominal distension, marked periumbilical tenderness, and rebound tenderness; fever, tachycardia, and normal blood pressure	Terminal ileum, 30 cm proximal to the ileocecal junction	Emergency midline laparotomy; thorn removed; primary repair of perforation with 4–0 PDS; uneventful recovery; discharged on postoperative day 8; well at 1-year follow-up
Fongfung et al. (2021) (29)	Thailand	Case report	A 70-year-old female	*Sandoricum koetjape* (Kathon) and mangosteen seeds	Abdominal pain and vomiting 2 h before admission; rebound tenderness at the lower abdomen with stable vital signs and no fever	Sigmoid colon and rectosigmoid junction	Abdominal exploration revealed seeds protruding through perforation; abscess in pelvic cavity; Hartmann’s operation performed; patient stable postoperatively
Naidu et al. (2020) (30)	Malaysia	Case report	A 70-year-old female	*Sandoricum koetjape* (Kathon) seeds	Lower abdominal pain for 3 days, diarrhea for 1 day with last episode associated with fresh blood per rectum, fever for 2 days; on examination: alert, pink, normal BP, tachycardia; lower abdominal tenderness and guarding (right iliac fossa), lateral rectal wall tenderness on DRE	Upper rectum (nearly half the bowel circumference)	Emergency laparotomy revealed pus in lower abdomen and three santol seeds protruding through perforation; localized fecal contamination; Hartmann’s procedure performed; patient recovered well and was discharged once oral intake resumed, ambulating, and able to manage colostomy
Changsrisuk, 2018 (31)	Thailand	A Retrospective Study	25 patients (14 males, 11 females); ages ranged from 31–90 years	*Sandoricum koetjape* (Kathon) seeds	Most presented with peritonitis (60.0%), others with peptic ulcer perforation (20.0%), appendicitis (8.0%), abdominal pain (8.0%), or gut obstruction (4.0%).	Sigmoid colon and rectosigmoid colon	Surgical treatments included Hartmann operation, repair with proximal loop colostomy, and colon resection with proximal loop colostomy; 24% mortality rate.
Changsrisuk and Chutipongtanate (2013) (32)	Thailand	A Retrospective Study	30 patients (14 males, 16 females), aged 42–85 years	*Sandoricum koetjape* (Kathon) seeds	Severe abdominal pain (80.0%), abdominal pain with diarrhea (16.7%), lower gastrointestinal bleeding (3.3%); all diagnosed intraoperatively with rectosigmoid perforation and seeds present at or near the perforation site	Rectosigmoid colon	Surgical intervention—Hartmann procedure (70%), repair with proximal loop colostomy (16.67%), resection and re-anastomosis with loop colostomy (13.33%); 20% mortality rate within 28 days, causes of death included septic shock with renal failure, septic shock with metabolic acidosis, and congestive heart failure with pulmonary edema.
Bell and Mustard, (1997) (33)	Canada	Case report	A 46-year-old male	Bay leaf (*Laurus nobilis*)	1-day history of nausea, abdominal discomfort, and right lower quadrant tenderness; provisional diagnosis of acute appendicitis	Meckel’s diverticulum	Exploratory surgery with McBurney incision, removal of normal appendix, wedge resection of inflamed Meckel’s diverticulum; smooth recovery and discharged on postoperative day 2

BP, blood pressure; bpm, beats per minute; DJ, duodenojejunal flexure; DRE, digital rectal examination; HR, heart rate; ICU, intensive care unit; mmHg, millimeters of mercury; PDS, polydioxanone suture; POD, postoperative day; R&A, resection and anastomosis; RIF, right iliac fossa; RLQ, right lower quadrant.

The differential diagnosis for RLQ pain considers clinical reasoning from the anatomic location and what etiologies would be most likely and includes acute appendicitis, infectious ileocecitis, right-sided diverticulitis, ureterolithiasis, and psoas or rectus muscle hematomas (12, 13). The patient’s clinical presentations were concerning for appendicitis, following a classic pattern: early-stage, unfocused abdominal pain at the umbilicus that eventually localized to the RLQ, accompanied by low-grade fevers and localized tenderness with rebound (14), which resulted in the investigation algorithm prioritizing appendicitis early in the work-up. However, abdominal computed tomography (CT) did not show free intraperitoneal air nor evidence of an ingested foreign body, demonstrating the limitations of imaging modalities in certain scenarios. Although CT is highly specific, reported sensitivities vary: sensitivity 50.0% and specificity 95.4% for free intraperitoneal air in blunt trauma (15); sensitivity 79.5% and specificity 96.4% for blunt small-bowel perforation (16); and pooled sensitivity 85.3% and specificity 96.1% for bowel/mesenteric injury (17). Therefore, clinical–imaging discrepancies necessitate ongoing awareness of uncommon causes.

Several practical considerations arise. First, although bowel perforation due to food ingestion is rare, it should remain in the differential diagnosis of acute RLQ pain. A targeted dietary history within the preceding 6–8 h may be informative in some cases; however, orocecal transit time (OCTT) should not be used to localize injury. OCTT reflects average transit physiology—typically 3.5–6.3 h and shortened by a subsequent meal at 180 min—but may not correspond to the timing or site of perforation because plant material can initially implant in the mucosa and progress to full-thickness injury over variable intervals (18, 19). In the present case, the patient’s last meal occurred approximately 7 h before symptom onset, but he did not report specific ingestion of *G. gnemon*; frequency, quantity, and preparation were not documented. Moreover, the terminal ileum is a plausible site for impaction due to regional angulation (20), further decoupling presentation from expected transit times. Second, safe food preparation practices—such as removing hard or fibrous stems—and thorough mastication are essential preventive measures. Proper chewing increases the surface area available for enzymatic digestion and reduces the likelihood of mucosal injury during peristalsis, thereby lowering the risk of food-associated mechanical perforation (21).

*Gnetum gnemon* var. *tenerum* is a tropical plant species native to India, Indonesia, and Thailand, and it is known locally as “liang” in southern Thailand (22–24). The tender leaves are often used in stir-fried preparations or coconut soup, while the fibrous side stalks are often discarded (25). In a study of insoluble fiber fractions in *G. gnemon* stems, it was shown that there is significantly more insoluble fiber (5–6 times more) than soluble fiber in the stems and similarly for intermediate leaves (23). This data supports a physiological plausibility whereby the stiff side stalks of *G. gnemon* would become angular, non-compressible fragments in the patient because the fibers were poorly chewed. Still, small-bowel perforation is rare in fibrous diets and is not suggested at the population level. Risk is likely more complicated within the combination of fragment rigidity/geometry, adequacy of mastication, and anatomic features such as terminal-ileal angulation. This context is therefore not advantageous for hypothesizing localization or risk but rather for counseling for the practical benefits of preparation (removing the tough stalks) and chewing (26).

Intraoperatively, one should take care to inspect adjacent bowel when the appendix does not appear concerning or is mildly inflamed. It has been reported that the negative appendectomy rate is 11.7%, with the negative appendectomy associated with prior abdominal surgery (odds ratio [OR], 2.75) and an Alvarado score less than 7 (OR 8.52) related to the absence of significant clinical or laboratory symptoms or findings (i.e., abdominal pain, rebound, leukocytosis, or neutrophilia) (27). The literature supports thorough exploration to avoid missed diagnoses. Thorough exploration may also lead to a change in management if non-appendiceal pathology is identified, as in our case.

This study had several limitations. First, as a single case, it cannot determine the incidence; additionally, plant-related bowel perforations may be under-identified, and further longitudinal or cross-sectional studies are warranted. Second, we followed our patient for a relatively short time (2 weeks), which limited our ability to comment on long-term sequelae such as strictures or recurrent obstruction. Third, while CT was performed, it was not optimized for foreign body visualization, and diagnostic accuracy, among other factors, may vary depending on the imaging protocols and the radiologist’s reading experience. Few studies have evaluated the pre-operative diagnostic accuracy in specific cases of plant-related perforations, emphasizing the need for more research in this area. Likewise, our discussion of fiber composition is provided to contextualize mechanistic plausibility only; detailed exposure data (frequency, quantity, preparation) were unavailable, and causal inference from composition alone is not possible. Finally, our discussion of OCTT is provided for physiologic context only; it has limited clinical utility for estimating injury location in suspected plant/foreign-body perforation and could not be applied to this case given the unavailable dietary details.

In conclusion, this case illustrates an uncommon terminal ileal perforation likely related to *G. gnemon* that clinically mimicked acute appendicitis. Notable findings from this case included the absence of preoperative CT “red flags” (e.g., no free intraperitoneal air and no visible foreign body on imaging), and the appendix appeared benign intraoperatively. These discordant findings illustrate the limitations of imaging, as well as the need to keep an open mind regarding diagnostic considerations and apply thorough dietary histories and thorough intraoperative examinations when presenting symptoms, imaging, and operative findings diverge from one another. Though the dietary practices vary globally, awareness of the regional consumption of fibrous edible plants may improve diagnostic suspicion and patient awareness in settings where these foods could be considered staples.

## Data Availability

The original contributions presented in the study are included in the article/Supplementary material, further inquiries can be directed to the corresponding author.

## References

[1] Pouli S Kozana A Papakitsou I Daskalogiannaki M Raissaki M. Gastrointestinal perforation: clinical and MDCT clues for identification of aetiology. Insights Imaging. (2020) 11:31. doi: 10.1186/s13244-019-0823-6, PMID: 32086627 PMC7035412

[2] Tan KK Bang SL Sim R. Surgery for small bowel perforation in an Asian population: predictors of morbidity and mortality. J Gastrointest Surg. (2010) 14:493–9. doi: 10.1007/s11605-009-1097-y, PMID: 19997984

[3] Lee DB Shin S Yang CS. Patient outcomes and prognostic factors associated with colonic perforation surgery: a retrospective study. J Yeungnam Med Sci. (2022) 39:133–40. doi: 10.12701/yujm.2021.01445, PMID: 34710320 PMC8913914

[4] Shin R Lee SM Sohn B Lee DW Song I Chai YJ . Predictors of morbidity and mortality after surgery for intestinal perforation. Ann Coloproctol. (2016) 32:221–7. doi: 10.3393/ac.2016.32.6.221, PMID: 28119865 PMC5256250

[5] Gebran A Proaño-Zamudio JA Argandykov D Dorken-Gallastegi A Renne AM Parks JJ . Association of Comorbidities and Functional Level with Mortality in geriatric bowel perforation. J Surg Res. (2023) 285:90–9. doi: 10.1016/j.jss.2022.12.027, PMID: 36652773

[6] Dursun A Oğuzdoğan GY Teker K Tuncer K. Isolated small bowel perforations: etiology and management. Turk J Colorectal Dis. (2022) 32:134–40. doi: 10.4274/tjcd.galenos.2021.2021-11-1

[7] Golash V Willson PD. Early laparoscopy as a routine procedure in the management of acute abdominal pain: a review of 1,320 patients. Surg Endosc Interv Tech. (2005) 19:882–5. doi: 10.1007/s00464-004-8866-1, PMID: 15920682

[8] Adisa AO Alatise OI Arowolo OA Lawal OO. Laparoscopic appendectomy in a Nigerian teaching hospital. J Soc Laparoendosc Surg. (2012) 16:576–80. doi: 10.4293/108680812X13462882737131, PMID: 23484567 PMC3558895

[9] Yogish V Grover H Bharath V. A comparative study between open appendicectomy and laparoscopic appendicectomy: a single-center experience. World J Laparosc Surg. (2021) 14:205–7. doi: 10.5005/jp-journals-10033-1468

[10] Mohanty D Dugar D Waliya A. A right-angled thorn in the bowel: a curious case of small bowel perforation. Cureus. (2023) 15:e44068. doi: 10.7759/cureus.44068, PMID: 37750116 PMC10517881

[11] Shafique MS Bhatti HW Farooqui MR Hanif M. EP112 - an unusual case of seed bezoars intestinal perforation. Br J Surg. (2024) 111:viii113–viii114. doi: 10.1093/bjs/znae197.442

[12] Amey L Donald KJ Teodorczuk A. Teaching clinical reasoning to medical students. Br J Hosp Med. (2017) 78:399–401. doi: 10.12968/hmed.2017.78.7.399, PMID: 28692355

[13] Mettarikanon D Tawanwongsri W. Analysis of patient information and differential diagnosis with clinical reasoning in pre-clinical medical students. Int Med Educ. (2024) 3:23–31. doi: 10.3390/ime3010003

[14] Moris D Paulson EK Pappas TN. Diagnosis and management of acute appendicitis in adults: a review. JAMA. (2021) 326:2299–311. doi: 10.1001/jama.2021.20502, PMID: 34905026

[15] Hefny AF Kunhivalappil FT Matev N Avila NA Bashir MO Abu-Zidan FM. Usefulness of free intraperitoneal air detected by CT scan in diagnosing bowel perforation in blunt trauma: experience from a community-based hospital. Injury. (2015) 46:100–4. doi: 10.1016/j.injury.2014.09.002, PMID: 25267401

[16] Park MH Shin BS Namgung H. Diagnostic performance of 64-MDCT for blunt small bowel perforation. Clin Imaging. (2013) 37:884–8. doi: 10.1016/j.clinimag.2013.06.005, PMID: 23886598

[17] Abdel-Aziz H Dunham CM. Effectiveness of computed tomography scanning to detect blunt bowel and mesenteric injuries requiring surgical intervention: a systematic literature review. Am J Surg. (2019) 218:201–10. doi: 10.1016/j.amjsurg.2018.08.018, PMID: 30201138

[18] Oyama T Fukuda S Shimoyama T Takahashi I Umeda T Danjo K . The oro-ileal transit of cellulose. J Food Sci. (2008) 73:H229–34. doi: 10.1111/j.1750-3841.2008.00942.x, PMID: 19021806

[19] Priebe MG Wachters-Hagedoorn RE Stellaard F Heiner AM Elzinga H Vonk RJ. Oro-cecal transit time: influence of a subsequent meal. Eur J Clin Investig. (2004) 34:417–21. doi: 10.1111/j.1365-2362.2004.01357.x, PMID: 15200493

[20] Hu T Zhang J Liu Y Chen L Cen W Wu W . Evaluation of the risk factors for severe complications and surgery of intestinal foreign bodies in adults: a single-center experience with 180 cases. Gastroenterol Rep. (2022) 10:goac036. doi: 10.1093/gastro/goac036, PMID: 35966628 PMC9366183

[21] Feron G Salles C. Food oral processing in humans: links between physiological parameters, release of flavour stimuli and flavour perception of food. Int J Food Stud. (2018) 7:1–12. doi: 10.7455/ijfs/7.1.2018.a1

[22] Shakya A Naorem A Khuraijam JS. *Gnetum* L., an underutilized plant of India: distribution and ethnobotany. Genet Resour Crop Evol. (2024) 71:29–38. doi: 10.1007/s10722-023-01704-7

[23] Siripongvutikorn S Usawakesmanee W Pisuchpen S Khatcharin N Rujirapong C. Nutritional content and microbial load of fresh Liang, *Gnetum gnemon* var. *tenerum* leaves. Foods. (2023) 12:3848. doi: 10.3390/foods12203848, PMID: 37893741 PMC10605991

[24] Wulandari C Sari DR Syahiib AN Novasari D. Correlation status of cultural significance index to characteristics of Krui indigenous people as a base for Repong Damar conservation efforts. Int J Des Nat Ecodyn. (2024) 19:69–80. doi: 10.18280/ijdne.190109

[25] Suksanga A Siripongvutikorn S Yupanqui CT Leelawattana R. The potential antidiabetic properties of Liang (*Gnetum gnemon* var. *tenerum*) leaves. Food Sci Technol. (2022) 42:e64522. doi: 10.1590/fst.64522

[26] Jahan K Qadri OS Younis K. Dietary Fiber as a functional food In: Ahmad S, Al-Shabib NA, editors. Functional food products and sustainable health. Singapore: Springer Nature Singapore Pte Ltd (2020). 155–67.

[27] Rodriguez-Garcia FA Rodríguez-Sánchez CE Naranjo-Chávez JC Torres-Ortiz-Ocampo CJ Rojas-Larios F Covarrubias-Ramírez K . Assessment of negative appendectomy in acute appendicitis diagnoses. Surg Pract Sci. (2025) 21:100281. doi: 10.1016/j.sipas.2025.100281, PMID: 40270918 PMC12017968

[28] Suárez-Gómez SA Velasco-Muñoz V Escobar-Castañeda F. When nature strikes back: understanding intestinal perforations caused by vegetable and animal bodies. Complications. (2024) 1:43–50. doi: 10.3390/complications1030008

[29] Fongfung MDS kaewpiboon MDG Suchato MDC. Colonic perforation due to phytozoars. Bangkok Med J. (2021) 17:69. doi: 10.31524/bkkmedj.2021.14.001

[30] Naidu R Muthusamy V Kamarulzaman M Zakaria A Khazim W Mohamed Kamil N. Rectal perforation caused by santol fruit seeds. Surg Chron. (2020) 25:284–5.

[31] Changsrisuk S. Sigmoid and rectosigmoid colon perforation with swallowed Santol seedChaoprayayomraj hospital. J Health Sci Thai. (2018) 17:SIV916–22.

[32] Changsrisuk S Chutipongtanate S. Risk-associated mortality in patients with peritonitis due to *Sandoricum koetjape* seed ingestion: a retrospective study. J Med Assoc Thai. (2013) 96:807–13. 24319851

[33] Bell CD Mustard RA. Bay leaf perforation of Meckel’s diverticulum. Can J Surg. (1997) 40:146–7. 9126131 PMC3952980

