# Supplementary material for: A sharp plant stem causing terminal ileal perforation with clinical presentation resembling acute appendicitis: a case report
Source: Front Med (Lausanne). 2025 Nov 28;12:1713022. doi: 10.3389/fmed.2025.1713022 (PMC12698559; doi:10.3389/fmed.2025.1713022)
Supplement: Supplementary file 1 [file Table_1.docx]

**Supplementary file 1**

**Supplementary Table 1.** Timeline of clinical course, investigations, and management

| **Time point** | **Event / Assessment** | **Key findings** | **Intervention / Outcome** |
| --- | --- | --- | --- |
| Day −1 (community) | Symptom onset | Dull, steady RLQ pain; low-grade fever; no nausea/vomiting/urinary symptoms | — |
| Day 0 (ED arrival) | Triage vitals | Temp 37.0 °C; BP 146/92 mmHg; HR 88 bpm; RR 20/min; SpO₂ 100% RA | Analgesia and standard ED care |
| Day 0 | Physical exam | Localized RLQ tenderness with rebound; no guarding; soft, non-distended abdomen | Surgical consult |
| Day 0 | Laboratory tests | WBC 14,170/µL (71% neutrophils); Hb 13.3 g/dL; Plt 355,000/µL; electrolytes/renal/coagulation within normal limits | — |
| Day 0 | Abdominal radiographs (acute series) | No pneumoperitoneum; no bowel obstruction | — |
| Day 0 | Contrast-enhanced CT | Appendix 7 mm; no focal mass or periappendiceal fat stranding; no free air/abscess; no foreign body visualized | Proceed to surgery based on clinical concern |
| Day 0 (OR) | Exploratory laparotomy via Lanz incision | 2.5 cm wooden foreign body in terminal ileum; localized perforation; minimal reactive peritoneal fluid; no cecal mass | Foreign body removal; primary repair of perforated ileum with interrupted sutures; appendectomy; EBL 10 mL |
| Post-op Day 0–2 | Inpatient recovery | Hemodynamically stable; no complications | Routine post-op care; diet advancement |
| Discharge (inpatient stay concluded) | Clinical status at discharge | Uncomplicated course | Discharged home |
| 2-week follow-up | Outpatient review | Asymptomatic; normal recovery | Routine follow-up as needed |

**Note.** CT, computed tomography; EBL, estimated blood loss; ED, emergency department; Hb, hemoglobin; OR, operating room; Plt, platelet count; RA, room air; RLQ, right lower quadrant; WBC, white blood cell count.
